# Supplementary material for: Characteristics of self-motion sensation after major earthquakes: An internet survey
Source: PLoS One. 2025 Sep 17;20(9):e0330450. doi: 10.1371/journal.pone.0330450 (PMC12443278; doi:10.1371/journal.pone.0330450)
Supplement: S1 File — Original language. (PDF) [file pone.0330450.s001.pdf]

質問紙

Q1) あなたの性別をお答えください。

☐ 男性　☐ 女性　☐ その他

Q2) あなたの年齢をお答えください。（半角数字でご記入ください）

Q3) あなたは、下記の既往歴や体質・性格がありますか？（いくつでも）

☐ めまいの治療をしたことがある（治療中を含む）

☐ 病院に行っていないが、めまいの経験がある

☐ 乗り物酔い、船酔いしやすい

☐ 頭痛持ちである

☐ かなり神経質

☐ 他の人よりも強く不安を感じやすい

☐ 上記に該当しない

Q4) 最初の地震はどこで経験されましたか？（県/市町村）

Q5) 最初の地震から今日までに、実際には地震ではないのにゆれやめまいを感じましたか？

☐ 10 回以上感じる

☐ 1〜9 回くらい感じる

☐ 一度も無い

Q6) そのゆれやめまい感は、初めて感じる感覚でしたか？

☐ 初めての感覚

☐ 以前に経験しためまいの病気に似ている

☐ めまいは地震の前からあり同じ程度

☐ めまいは地震の前からあり悪化

Q7) 1 回のゆれやめまい感の長さはどのくらいでしたか？

☐ 1 分未満

☐ 1〜5 分未満

☐ 5〜10 分未満

☐ 10〜30 分未満

☐ 30 分〜2 時間程度

☐ 半日程度

☐ ほぼ 1 日中

Q8) ゆれやめまいで日常生活に支障を感じたり困ったりしましたか？

☐ 困らない

☐ あまり困らない

☐ 少し困る

☐ 大いに困る

Q9) 現在、ゆれやめまいで不安や怖さを感じていますか？

☐ 不安や怖さを感じない

☐ 不安を感じる

☐ 怖さを感じる

☐ 不安であり怖い

Q10) そのゆれやめまいは、どのような感覚・症状でしたか？

合わせて起こる症状はありますか？（いくつでも）

☐ 身体が揺れる感じ

☐ 足もとがぐらつく

☐ 歩きにくい

☐ 景色がぐるぐる回る

☐ 頭を動かすと景色が回る

☐ 聞こえが悪くなった

☐ 耳鳴りがする

☐ 頭痛がする

☐ 吐き気がする

☐ 実際に嘔吐した

☐ その他 (自由記載)

Q11) そのゆれやめまいは、どのような時におきましたか？（いくつでも）

☐ 立っている時

☐ 歩いている時

☐ イスなどに座っている時

☐ 寝ている時

☐ 乗り物の中

☐ 頭を振ったり、動かした時

☐ どんな姿勢でもおこる

☐ その他 (自由記載)

Q12) そのゆれやめまいは、何日くらい続きましたか？

☐ 1 週間以内で消失

☐ 1～2 週間以内で消失

☐ 2 週間以上で消失

☐ いまだにあるが軽くなっている

- ☐ いまだに変わらない
- ☐ 悪くなっている

ご協力ありがとうございました
