# Supplementary material for: Characteristics of self-motion sensation after major earthquakes: An internet survey
Source: PLoS One. 2025 Sep 17;20(9):e0330450. doi: 10.1371/journal.pone.0330450 (PMC12443278; doi:10.1371/journal.pone.0330450)
Supplement: S2 File — (DOCX) [file pone.0330450.s002.docx]

Questionnaire

Q1) Sex

☐ Male　☐ Female　☐ Other

Q2) Age

Q3) Do you have any of the following signs or symptoms? (multiple responses allowed)

☐ I have been treated for dizziness (including during treatment)

☐ I have had dizziness but I have not been to a hospital.

☐ I am prone to motion sickness and/or seasickness.

☐ I frequently suffer from headaches.

☐ I am very nervous and/or sensitive.

☐ I tend to feel anxiety more strongly than others

☐ I do not fall into any of the above categories.

Q4) Where were you during the earthquake on January 1st? (Prefecture/city)

Region:

Q5) After the earthquake on January 1st, have you felt swaying or dizziness even though there was no actual earthquake at that time?

☐ More than 10 times

☐ 1-9 times

☐ Never

Q6) Was that the first time you felt that swaying or dizziness sensation?

☐ Yes, sensation experienced for the first time.

☐ No, this is similar to the dizziness that I experienced before.

☐ No, dizziness was present before the earthquake and remained at the same level.

☐ No, dizziness was present before the earthquake and had worsened.

Q7) How long did each swaying event or episode of dizziness last?

☐ Less than 1 minute

☐ Less than 1-5 minutes

☐ Five to 10 minutes

☐ 10 to 30 minutes

☐ 30 minutes to 2 hours

☐ About half a day

☐ Almost all day

Q8) Have you experienced disturbance in your daily life because of swaying or dizziness?

☐ None at all

☐ Not much

☐ Somewhat disturbed

☐ Much disturbed

Q9) Do you feel anxious or scared due to swaying or dizziness?

☐ I do not feel anxious or scared

☐ I feel anxious

☐ I feel scared

☐ Anxious and scared

Q10) What sensations/symptoms of swaying and dizziness did you experience?

Are there any other symptoms that occur in conjunction with these symptoms?" (multiple responses allowed)

☐ Feeling of body swaying

☐ Feeling wobbly underfoot

☐ Difficulty walking

☐ Feeling of the view spinning around in circles.

☐ Feeling of the view spinning when the head is moved.

☐ Hearing has become worse

☐ Tinnitus

☐ Headache

☐ I feel nauseous

☐ Vomiting

☐ Other

Q11) When did you feel the swaying or dizziness? (multiple responses allowed)

☐ While standing

☐ While walking

☐ While sitting on a chair

☐ While lying down

☐ In a vehicle

☐ Shaking or moving one’s head

☐ In any posture

☐ Other

Q12) How long did you feel swaying or dizziness?

☐ Disappeared within 1 week

☐ Disappeared within 1–2 weeks

☐ Disappeared after more than two weeks

☐ Still present but weakening

☐ Still the same

☐ Worsening

Thank you for your cooperation.
