# Supplementary material for: Characteristics of self-motion sensation after major earthquakes: An internet survey
Source: PLoS One. 2025 Sep 17;20(9):e0330450. doi: 10.1371/journal.pone.0330450 (PMC12443278; doi:10.1371/journal.pone.0330450)
Supplement: Attachment — Submitted filename: Comments.docx [file pone.0330450.s004.docx]

**“Characteristics of Self-Motion Sensation after Major Earthquakes: An Internet Survey” by Reiko Tsunoda et al.**

The effect of a large earthquake on people’s psychology and their daily life is the main topic which needs to pay more attentions. In this paper, the authors cast an internet survey on self-motion sensation by a questionnaire after the large 2024 7.6 earthquake in Noto Peninsula. 1000 responses have been received finally. They do some statistical work on the data and get some valuable results.

However, there are some uncertainties in the work:

Firstly of all, for a statistical investigation, the number of the 1000 samples in this time is too small to get a convincing result. Secondly, for a research paper, the investigating method (or technique) and the aim (or to solve a problem) are important. However, we cannot find any relevant contents in this paper. So this paper seems like a survey report instead of a research paper. Not mention that all figures are too simple and with low resolution. So a large amount of samples and scientific information are needed to support to this work if it is to publish in the future.
