# Supplementary material for: Characteristics of self-motion sensation after major earthquakes: An internet survey
Source: PLoS One. 2025 Sep 17;20(9):e0330450. doi: 10.1371/journal.pone.0330450 (PMC12443278; doi:10.1371/journal.pone.0330450)
Supplement: Attachment — Submitted filename: Response to Reviewers.docx [file pone.0330450.s006.docx]

We sincerely appreciate the reviewers’ insightful comments and suggestions, which have significantly improved the quality of our manuscript. In response to these revisions, the Abstract has also been updated accordingly.

**Reviewer #1:** Overall Assessment:
This study investigates the prevalence, characteristics, and prognostic factors of self-motion sensation (SMS) following the 2024 Noto Peninsula earthquake using an Internet survey. The topic is timely and relevant, particularly for disaster-affected regions. The manuscript is well-structured, and the methodology is appropriate for the research question. However, several issues need addressing before publication.

Major Comments:
1. Sample Representativeness and Bias:
o The participants were younger than the general population in the affected area, which may introduce selection bias. The authors acknowledge this limitation but should discuss its potential impact on the generalizability of the findings more thoroughly.

o The Internet survey method may exclude older or less tech-savvy individuals. Suggest including a comparison with demographic data from the affected regions to better contextualize the sample.

**Response 1**: Our data showed that younger age was significantly associated with impaired daily functioning owing to self-motion sensation. Prior studies (Nomura et al. 2014 and Miwa et al. 2021) also reported a high incidence of post-earthquake dizziness among younger to middle-aged adults. However, as the reviewer suggested, the affected area has an aging population, and the Internet survey method may exclude older or less tech-savvy individuals. The age distribution of the affected regions based on census data by region and the age distribution of the participants were added to the revised manuscript as Table 1. This comparison was also included in the Discussion section to further clarify our findings. Similarly, the limitations and potential impact of age bias on generalizability were further discussed.

Line 118

In the survey area, the proportion of residents aged ≥70 years was high, and the data did not follow a normal distribution. The median age of the participants was significantly younger than that of the residents in the target area (median test, P=0.000, Table 1).

Table 1. Age distribution.

Line 270

However, this strength is also a source of potential selection bias, a key limitation affecting the generalizability of Internet survey results [24]. Older adults, in particular, may be underrepresented owing to lower levels of digital literacy [25]. Our sample skewed younger than the general population of the affected area, which includes many individuals aged >70 years. While previous studies have shown that post-earthquake dizziness is more common in younger to middle-aged adults [8,9], future research should incorporate older individuals. Mixed-method approaches, such as caregiver-assisted surveys in shelters or nursing homes, may help mitigate this bias; however, they could introduce third-party influence [26].

Line 301

Third, selection bias was possible owing to the underrepresentation of older adults.

2. Terminology and Definitions:
o The term "self-motion sensation (SMS)" is introduced to distinguish the phenomenon from dizziness or vertigo. While the rationale is clear, the authors should justify this choice further by referencing additional literature or clinical guidelines.

o Clarify whether SMS is a novel classification or aligns with existing vestibular disorder frameworks (e.g., Persistent Postural-Perceptual Dizziness, PPPD).

**Response 2:** Self-motion sensation (SMS) is used as a phenomenological term to describe the experience of swaying or motion perception after an earthquake. We do not consider SMS to be a pathological condition, as more than half of the individuals who experienced it reported spontaneous resolution within 2 weeks. Thus, we used SMS as a descriptive term rather than a diagnostic label. However, in cases where SMS persists beyond 2 months or causes significant disruption to daily life, we believe it may represent a clinical condition that requires appropriate medical evaluation or intervention. It differs from “dizziness,” which, per the Bárány Society, implies impaired spatial orientation without motion perception. SMS also differs from Persistent Postural-Perceptual Dizziness (PPPD) in chronicity and criteria. We have expanded the explanation of self-motion sensation (SMS) in the Introduction and Discussion.

Line 53

Many individuals have reported brief episodes of dizziness occurring in the absence of actual aftershocks [8,9]. However, it remains unclear whether this phenomenon constitutes a pathological condition. Moreover, the symptoms reported varied across different studies [2,8,9,15], and its prognosis and the necessity of medical intervention remain partially elucidated.

Line 63

This study aimed to identify the characteristics of new-onset swaying or dizziness —defined here as self-motion sensation (SMS) — experienced after a major earthquake. We also sought to determine the factors associated with persistent symptoms or disturbance in daily life. Identifying these factors may inform early intervention strategies and support services for individuals at risk of persistent symptoms or functional impairment.

Line 189

we used the term SMS to describe the subjective experience of swaying or motion in the absence of seismic activity.

Line 193

Notably, most symptoms resolved within 2 weeks, suggesting that SMS is not inherently pathological.

Line 249

A key question is whether SMS may progress into chronic vestibular conditions such as Persistent Postural-Perceptual Dizziness (PPPD). Risk factors for SMS persistence overlap with those for PPPD. Although PPPD typically worsens with upright posture and motion, while SMS usually occurs during rest, both involve altered vestibular perception.

3. Longitudinal Data and Chronicity:
o The study is cross-sectional, with data collected at a single time point (two months post-earthquake). A follow-up study or analysis of chronic cases (e.g., symptoms persisting beyond three months) would strengthen the conclusions about prognosis.

o Consider discussing the potential progression of SMS to chronic vestibular disorders, such as PPPD, and its implications for clinical management.

**Response 3**: We agree with your observation. We have further explored the need for follow-up and longitudinal studies. We added a paragraph discussing the potential evolution of SMS to chronic conditions such as PPPD including supporting literature and implications for follow-up care.

Line 252

Longitudinal studies are required to clarify potential transitions. Early identification of individuals at risk may enable timely preventive interventions. Such studies should include ethically designed follow-up systems, and participants experiencing worsening symptoms should be referred to appropriate in-person psychological care.

Line 302

Finally, the long-term trajectory of SMS remains unclear. Future longitudinal research with repeated assessments is required to clarify whether SMS represents an early stage of chronic dizziness conditions such as PPPD.

Line 311

Our findings highlight the significance of early identification and support for individuals at risk of symptom persistence, as well as the importance of longitudinal studies in clarifying the potential progression of SMS to chronic vestibular disorders such as PPPD.

4. Anxiety as a Confounding Factor:
o Anxiety is identified as a key factor linked to prolonged SMS and daily life disruption. However, the study does not measure anxiety levels quantitatively (e.g., using standardized scales). Recommend citing literature on anxiety-vestibular interactions or suggesting future studies incorporate psychological assessments.

**Response 4:** As observed by the reviewers, it is preferable to evaluate the respondents using standardized anxiety measures such as HADS and STAI; however, as this was an online survey targeting the general public in the affected areas, we minimized the number of questions and the burden of responses. We acknowledge this limitation and added a note recommending the use of standardized anxiety measures (e.g., HADS and STAI) in future research. Relevant literature linking vestibular symptoms and anxiety has been cited.

Line 212

These findings are consistent with those of prior studies. Kumar et al. reported frequent comorbid anxiety, panic, agoraphobia, and psychosomatic traits in patients with post-earthquake balance disorders [2]. Eker et al. found significantly elevated anxiety and depression scores among earthquake-affected individuals, as measured using the Hospital Anxiety and Depression Scale [10]. Homma et al. identified that high state anxiety scores, as measured using the State-Trait Anxiety Inventory, were significantly associated with equilibrium dysfunction in individuals exposed to aftershocks [18]. Çelebi et al., in a long-term follow-up conducted 1 year after the earthquake, further demonstrated that trait anxiety — rather than state anxiety — was more predictive of persistent symptoms [12]. While our findings are consistent with these observations, they should be interpreted with caution. The cross-sectional design limits causal inference, and we did not use a standardized anxiety scale. Future research should consider validated instruments such as the Hospital Anxiety and Depression Scale or State-Trait Anxiety Inventory to better evaluate psychological factors.

5. Clinical Implications:
o The discussion of "non-spinning vertigo" as a potential pathological outcome is intriguing but speculative. Provide more evidence or references to support this classification.

o Include practical recommendations for healthcare providers in disaster settings (e.g., screening for anxiety in individuals reporting SMS).

**Response 5:** As you have observed, the expression gave the impression that SMS is a pathological symptom with the characteristics of non-spinning vertigo. We have deleted “non-spinning vertigo” from the article. We have added to our discussion the immediacy of benefits for survey participants and affected areas.

Line 287

This study underscores the significance of addressing anxiety and fear elicited by SMS — a form of state anxiety specifically associated with earthquake-related symptoms. Accordingly, post-disaster surveys should be designed not merely as research instruments but as tools that provide direct, meaningful benefits to participants. Such surveys should aim to collect data and serve as gateways to support systems by offering accurate health information, screening for psychological symptoms, and facilitating access to mental health services. Achieving this goal requires coordinated efforts among researchers, clinicians, and local authorities [28]. Remote consultation systems involving professionals outside the disaster-affected area may further help alleviate the burden on local healthcare infrastructure while maintaining continuous support for those affected.

Minor Comments:
1. Figures and Tables:
o Tables 1–3 are well-presented, but consider simplifying the categorization explanations (e.g., age groups) for clarity.

**Response 1**: The explanation has been simplified.
Table 2,3

Coding of independent variables:
regions (1 = Ishikawa, 2 = Toyama, 3 = Fukui), sex (1 = male, 0 = female), age group (0 = ≤35 years, 1 = 36–50 years, 2= 51–65 years, 3= ≥66 years), and other factors (1 =yes, 0 =no).

2. Language and Clarity:
o The manuscript is generally well-written, but some sentences are overly complex (e.g., the Abstract’s conclusion). Minor editing for conciseness is recommended.
o Define abbreviations (e.g., SMS) at first use in the Abstract and Introduction.

**Response 2**: In response to the reviewers' suggestions, we carefully requested English proofreading and revised the text to be concise and clear. We defined abbreviations (e.g., SMS) at first use in the Introduction. As the journal does not allow abbreviations in the Abstract, we have avoided using them there.

3. References:

o The literature review is comprehensive, but some citations (e.g., media reports) could be replaced with peer-reviewed studies where possible.

**Response 3**: The latest literature on dizziness after earthquakes has been added to the references, and media reports have been deleted.

Recommendation:
Major Revision Required. The manuscript addresses an important gap in disaster-related health research but requires revisions to address the above concerns, particularly regarding sample bias, terminology, and clinical implications. With these revisions, the study would be a valuable contribution to PLOS ONE.

Additional Suggestions:
• Include a brief paragraph on the cultural context of "earthquake drunk" in Japan to aid international readers.

**Response:** The following text has been added.

Line 46

In Japan, the colloquial term jishin-yoi ("earthquake drunk") became common after the Great East Japan Earthquake on March 11, 2011. By April of that year, it was frequently referenced in otolaryngology clinical reports. In 2014, Nomura et al. formally defined this phenomenon as Post-Earthquake Dizziness Syndrome (PEDS) [8]. Subsequent studies, including a population-based survey following the 2016 Kumamoto earthquake in Japan [9] and multicenter investigations after the 2023 Kahramanmaraş earthquakes in Türkiye [10–14], have further documented PEDS in various seismic settings.

• Discuss the ethical considerations of Internet surveys in post-disaster settings in more depth.

**Response:** Thank you for this important suggestion. We have expanded our discussion on the ethical considerations related to conducting internet-based surveys in post-disaster settings.

Line 260

**Minimizing psychological burden**: Individuals in the early aftermath of a disaster are usually coping with trauma, loss, and severe disruption. Internet surveys enable voluntary, anonymous participation at the respondent’s own pace and in their preferred setting.

**Avoiding interference with recovery efforts**: On-site surveys conducted in evacuation shelters or disaster areas may disrupt relief operations. Internet-based surveys eliminate the need for physical presence and reduce this risk.

**Ensuring autonomy and ethical compliance:** Participants can receive detailed information about study aims, data handling, and confidentiality, and consent can be obtained via a digital opt-in procedure.

**Enhancing accessibility and equity**: Internet-based surveys enable rapid, wide-reaching data collection across dispersed populations. However, this strength is also a source of potential selection bias, a key limitation affecting the generalizability of Internet survey results [24]. Older adults, in particular, may be underrepresented owing to lower levels of digital literacy [25]. Our sample skewed younger than the general population of the affected area, which includes many individuals aged >70 years. While previous studies have shown that post-earthquake dizziness is more common in younger to middle-aged adults [8,9], future research should incorporate older individuals. Mixed-method approaches, such as caregiver-assisted surveys in shelters or nursing homes, may help mitigate this bias; however, they could introduce third-party influence [26].

Final Note: The authors are commended for their timely research and clear presentation. Addressing these points will enhance the manuscript’s impact and readiness for publication.　
________________________________________
Reviewer Decision: Revise and Resubmit

**Reviewer #2:** This manuscript presents a cross-sectional internet survey investigating self-motion sensations (SMS) following the 2024 Noto Peninsula earthquake in Japan. While the topic is relevant and timely, there are several significant concerns that should be addressed before publication.
Major Concerns
**Comments 1.** The internet survey methodology introduces significant selection bias, particularly evident in the younger demographic skew of respondents. While acknowledged, this limitation warrants more extensive discussion regarding its impact on the validity and generalizability of findings.

**Response 1:** Our data showed that younger age was significantly associated with impaired daily functioning owing to self-motion sensation. Prior studies (Nomura et al. 2014, Miwa et al. 2021) also reported a high incidence of post-earthquake dizziness among younger to middle-aged adults. However, as the reviewer suggested, the affected area has an aging population, and the Internet survey method may exclude older or less tech-savvy individuals. The age distribution of the affected regions based on census data by region and the age distribution of the participants were added to the revised manuscript as Table 1. We have expanded the discussion of selection bias and its implications for generalizability.

Table 1. Age distribution.

Line 270

However, this strength is also a source of potential selection bias, a key limitation affecting the generalizability of Internet survey results [24]. Older adults, in particular, may be underrepresented owing to lower levels of digital literacy [25]. Our sample skewed younger than the general population of the affected area, which includes many individuals aged >70 years. While previous studies have shown that post-earthquake dizziness is more common in younger to middle-aged adults [8,9], future research should incorporate older individuals. Mixed-method approaches, such as caregiver-assisted surveys in shelters or nursing homes, may help mitigate this bias; however, they could introduce third-party influence [26]. Additionally, self-selection bias should be considered, as participants may be drawn to the study based on personal interest or incentives [24].

Line 301
Third, selection bias was possible owing to the underrepresentation of older adults.

**Comments** **2.** The absence of a control group makes it impossible to determine whether SMS was actually caused by the earthquake rather than other factors.

**Response 2**: We acknowledge the absence of a control group as a limitation. Owing to the nature of disaster-related surveys, obtaining an unaffected control population under the same conditions was not feasible. We added this point to the limitation.

Line 297

First, the cross-sectional design and lack of a non-exposed control group precluded causal inference. Although associations between SMS and factors such as anxiety or proximity to the epicenter were identified, these should be interpreted with caution.

**Comments** **3.** The 2-month delay between the earthquake and data collection introduces substantial recall bias, especially for symptoms that may have resolved quickly.

**Response 3:** We clarified that the survey timing (2 months post-event) was chosen to avoid undue burden during the acute disaster phase. While recall bias is acknowledged, our use of uniform timing across participants improves internal consistency. This explanation has been expanded in the Methods and Discussion sections.

Line 84

To maintain internal consistency across participants, the response window was limited to 24 h, and 1,000 responses were obtained.

Line 281

The survey was conducted 2 months after the earthquake, during a transitional period from the acute to the recovery phase, when many individuals had begun to restore daily routines [27]. Although recall bias was possible, particularly given that most SMS cases resolved within 2 weeks, this timing was considered ethically appropriate and allowed for an accurate reporting of persistent cases. The 24-h response window further ensured internal consistency and provided a high-resolution cross-sectional snapshot..

Line 300

Second, the 2-month gap between the earthquake and the survey may have introduced recall bias, particularly for participants whose symptoms resolved quickly.

**Comments 4.** The cross-sectional design cannot establish causality between anxiety and SMS persistence/disruption, yet causal language is sometimes implied in the discussion.

**Response 4**: We have carefully revised the manuscript to remove any language suggesting causality between anxiety and SMS, consistently referring to associations only.
Line 220

While our findings are consistent with these observations, they should be interpreted with caution. The cross-sectional design limits causal inference, and we did not use a standardized anxiety scale.

Line 297

First, the cross-sectional design and lack of a non-exposed control group precluded causal inference. Although associations between SMS and factors such as anxiety or proximity to the epicenter were identified, these should be interpreted with caution.

**Comments 5.** The authors fail to distinguish between pre-existing anxiety versus earthquake-induced anxiety, which could significantly confound the results.

**Response 5:** It is preferable to evaluate the respondents using standardized anxiety measures such as HADS and STAI; however, as this is an online survey targeting the general public in the affected areas, we minimized the number of questions and the burden of responses. Therefore, we want to clarify that our questionnaire explicitly distinguished between pre-existing anxiety (trait anxiety) and earthquake-induced anxiety using two items:

Q3: “Do you have any of the following signs or symptoms? — I tend to feel anxiety more strongly than others.” → This question was used to assess pre-existing trait anxiety.

Q9: “Do you feel anxious or scared due to swaying or dizziness?” → This item captured a state of anxiety specifically related to earthquake-triggered symptoms.

In the statistical analysis, both variables were treated separately to examine their respective associations with persistence and disruption of self-motion sensation (SMS). We have revised the Methods and Discussion sections to more clearly state this distinction and how it was addressed analytically. We appreciate the opportunity to clarify this important point.

Line 85

The questionnaire was used to collect information on age, sex, geographic location during the earthquake, new-onset swaying or dizziness, concomitant symptoms, individual characteristics (e.g., history of dizziness, motion sickness, and trait anxiety), the impact of symptoms on daily life, emotional responses (e.g., anxious or scared), and symptom duration (S1 and S2 Files).

Tables 2 and 3

Analysis distinguished between “Trait anxiety” and “Feeling anxious or scared owing to SMS” (refers to state anxiety)

Line 161

Logistic regression analysis indicated younger age (OR: 0.588; 95% confidence interval (CI): 0.403–0.857; E-value = 1.93) and feeling anxious or scared owing to SMS (OR: 12.176; 95% CI: 5.574–26.598; E-value = 6.44) as significant factors associated with daily life disturbance (Table 2).

L173

Logistic regression analysis showed that proximity to the epicenter (OR: 0.638; 95% CI: 0.436 – 0.933; E-value = 1.81) and feeling anxious or scared owing to SMS (OR: 3.668; 95% CI: 2.202 – 6.110; E-value = 3.24) were significantly associated with symptom persistence (Table 3).

Line 207

Our analysis showed that younger age and emotional responses such as feeling anxious or scared owing to SMS were significantly associated with disturbance in daily functioning. Additionally, symptom persistence was associated with proximity to the epicenter and emotional responses. In our study, “feeling anxious or scared owing to SMS” reflects a form of state anxiety specifically related to earthquake-triggered symptoms.

**Comments** 6. The study lacks sensitivity analyses to evaluate the robustness of the findings.

**Response 6:** To evaluate the robustness of our findings against unmeasured confounding, we conducted a sensitivity analysis using the E-value, which quantifies the minimum strength of association that an unmeasured confounder would need to have with the exposure and the outcome to fully explain the observed association. The calculated E-values suggested that our findings were reasonably robust to potential unmeasured confounding. We have added this analysis to the Results.

Line 108

Sensitivity analysis using E-values was conducted to assess the potential influence of unmeasured confounding factors [16].

Line 161

Logistic regression analysis indicated younger age (OR: 0.588; 95% confidence interval (CI): 0.403–0.857; E-value = 1.93) and feeling anxious or scared owing to SMS (OR: 12.176; 95% CI: 5.574–26.598; E-value = 6.44) as significant factors associated with daily life disturbance (Table 2).

L173

Logistic regression analysis showed that proximity to the epicenter (OR: 0.638; 95% CI: 0.436 – 0.933; E-value = 1.81) and feeling anxious or scared owing to SMS (OR: 3.668; 95% CI: 2.202 – 6.110; E-value = 3.24) were significantly associated with symptom persistence (Table 3).

**Comments 7.** The operational definition of SMS requires refinement. The distinction between SMS and aftershocks perception is inadequately addressed.
Given that proximity to epicenter was identified as a risk factor for prolonged symptoms, it is crucial to explain how participants distinguished between SMS and actual aftershocks, which were common in this region.

**Response 7:** The questionnaire asked, “Have you felt swaying or dizziness even though there was no actual earthquake at that time?” Therefore, it was assumed that when participants felt the SMS, they visually checked whether the surrounding objects were swaying or checked the earthquake report on the Internet. However, as you have observed, the possibility that they were sensitive to aftershocks cannot be ruled out. This point was also clearly stated in the discussion.

Line 229

Following strong vestibular stimulation, such as that caused by an earthquake, heightened sensory vigilance may activate a fight-or-flight response. While sensory reweighting facilitates adaptation, it could be maladaptive, leading to persistent or exaggerated perceptions of motion even in the absence of actual movement [15, 19]. In hypersensitive individuals, even minimal aftershocks may be perceived as significant.

**Comments 8.** There is no clear operational definition distinguishing "self-motion sensation" from "dizziness," despite the attempt to discuss this in lines 176-185. This discussion lacks necessary rigor.

**Response 8**: Self-motion sensation (SMS) is used as a phenomenological term to describe the experience of swaying or motion perception after an earthquake. We do not consider SMS to be a pathological condition, as more than half of the individuals who experienced it reported spontaneous resolution within 2 weeks. Thus, we used SMS as a descriptive term rather than a diagnostic label. We have expanded the explanation of the relationship with PESD in previous studies in the Discussion.

Line 188

While previous studies referred to this condition as PEDS [8,15] or post-earthquake dizziness [9], we used the term SMS to describe the subjective experience of swaying or motion in the absence of seismic activity. SMS involved brief episodes of body sway lasting less than 1 min and occurred primarily while sitting or standing still without rotational vertigo or head movement. According to the Bárány Society [17], “dizziness” refers to disturbed spatial orientation without a false perception of motion, making it a less precise term for describing SMS. Notably, most symptoms resolved within 2 weeks, suggesting that SMS is not inherently pathological. In contrast, some studies have reported more persistent and complex symptoms following earthquakes. For example, Kumar et al. documented cases of continuous ground shaking, sustained fear, and imbalance. Symptoms included lightheadedness, swinging sensations, headaches, blackouts, and loss of consciousness [2]. Miwa et al. described PEDS as encompassing dizziness, vertigo, imbalance, nausea, and a persistent sensation of motion, typically triggered soon after the earthquake [15]. These findings suggest that symptoms evolve over time and that SMS progresses into conditions that meet diagnostic criteria for vestibular or neuropsychiatric disorders. As our study was conducted 2 months after the earthquake and involved a tightly controlled 24-h response window, it enabled a clear cross-sectional characterization of SMS during a relatively stable recovery phase.

**Comments 9.** The paper lacks sufficient discussion of the physiological mechanisms underlying SMS. A more robust theoretical framework connecting vestibular function, anxiety, and seismic experiences would strengthen the manuscript.

**Response 9:** We added a section reviewing theories on vestibular-somatosensory integration and neural adaptation following extreme sensory input, referencing relevant vestibular and psychosomatic literature.

Line 225

The underlying mechanisms of SMS and the progression of its symptoms remain speculative. However, neural adaptation processes and vestibular system responses under extreme conditions such as earthquakes may play a role.

**Sensory reweighting:** The human vestibular system integrates visual, proprioceptive, and vestibular inputs. Following strong vestibular stimulation, such as that caused by an earthquake, heightened sensory vigilance may activate a fight-or-flight response. While sensory reweighting facilitates adaptation, it could be maladaptive, leading to persistent or exaggerated perceptions of motion even in the absence of actual movement [15, 19]. In hypersensitive individuals, even minimal aftershocks may be perceived as significant.

**Prediction errors and mismatch processing:** Earthquakes generate discrepancies between expected and actual sensory input, disrupting the brain’s internal models of motion and spatial orientation. These prediction errors may contribute to prolonged sensations of motion or postural instability [20].

**Psychophysiological interplay:** Anxiety and hypervigilance may amplify somatic sensations and impair vestibular compensation, sustaining SMS perception [21].

**Central sensitization and cortical plasticity:** Repetitive or intense vestibular input during earthquakes may cause maladaptive neuroplastic changes in the cortical regions responsible for spatial orientation. Functional imaging studies have implicated the temporoparietal junction and insular cortex in persistent dizziness and spatial disorientation [22, 23].

Peripheral vestibular dysfunction may also play a role. Strong shaking during earthquakes may cause inertial stress on otoliths in the utricle, leading to otolith dysfunction [1]. Repeated shaking could result in otolith detachment, contributing to vestibular impairment or benign paroxysmal positional vertigo [3,4]. Eker et al. reported dysfunction in the left posterior semicircular canal among patients experiencing post-earthquake dizziness [10].

**Comments 10.** Multiple comparisons were conducted without appropriate correction methods, raising concerns about Type I error.

**Response 10:** To account for multiple testing in the two primary outcomes, Bonferroni correction was applied and the analysis was repeated.

Line 106
To adjust for multiple testing across the two primary outcomes, Bonferroni correction was applied by multiplying P-values by two, and statistical significance was defined as a Bonferroni-adjusted P-value <0.05.

Minor Concerns
**Comments 11.** There is an obvious grammatical error in lines 161-162: "...excluded from the study. that the results show that 43% of those who experienced a major earthquake also experienced an SMS." The sentence is fragmented and needs restructuring.

**Response 11:** We have corrected the following

Line 179

We focused specifically on newly developed swaying or dizziness symptoms, referred to as SMS, and excluded participants who reported only a worsening of pre-existing dizziness. Our findings revealed that 43% of participants experienced such sensations.

**Comments 12.** Several sentences are excessively long and complex (e.g., lines 115-118), reducing readability. These should be divided into shorter, clearer statements.

**Response 12:** The entire text was proofread in English to improve the grammatical flow.

Line 123

After the January 1 earthquake, 416 of the 968 participants reported a swaying or dizziness sensation in the absence of actual seismic activity. They confirmed this as a novel sensation.

**Comments 13.**Terminology inconsistency exists between "disrupting daily life" in the abstract and "disturbing daily life" in the main text.

**Response 13:** Terminology has been standardized throughout the manuscript. Standardized to “disturbance in daily life”.

**Comments 14.** Some important conclusions lack direct citation support, such as the discussion of SMS characteristics in lines 173-180.

**Response 14:** We thank the reviewers for their comments on the terminologies used in this study. As mentioned above, we chose the term “self-motion sensation (SMS)” to directly reflect the subjective experiences reported by the participants. We do not consider SMS to be a pathological condition but rather use it as a descriptive term. Previous studies have reported vestibular impairments that differ from SMS. We discussed the timing of the study and the possibility of changes in SMS over time.

Line 188

While previous studies referred to this condition as PEDS [8,15] or post-earthquake dizziness [9], we used the term SMS to describe the subjective experience of swaying or motion in the absence of seismic activity. SMS involved brief episodes of body sway lasting less than 1 min and occurred primarily while sitting or standing still without rotational vertigo or head movement. According to the Bárány Society [17], “dizziness” refers to disturbed spatial orientation without a false perception of motion, making it a less precise term for describing SMS. Notably, most symptoms resolved within 2 weeks, suggesting that SMS is not inherently pathological. In contrast, some studies have reported more persistent and complex symptoms following earthquakes. For example, Kumar et al. documented cases of continuous ground shaking, sustained fear, and imbalance. Symptoms included lightheadedness, swinging sensations, headaches, blackouts, and loss of consciousness [2]. Miwa et al. described PEDS as encompassing dizziness, vertigo, imbalance, nausea, and a persistent sensation of motion, typically triggered soon after the earthquake [15]. These findings suggest that symptoms evolve over time and that SMS progresses into conditions that meet diagnostic criteria for vestibular or neuropsychiatric disorders.

**Comments 15.** Provide an in-depth discussion of neural adaptation mechanisms and vestibular system responses to extreme experiences.

**Response 15:** We have expanded the discussion on the mechanisms of SMS.

Line 225

The underlying mechanisms of SMS and the progression of its symptoms remain speculative. However, neural adaptation processes and vestibular system responses under extreme conditions such as earthquakes may play a role.

**Sensory reweighting:** The human vestibular system integrates visual, proprioceptive, and vestibular inputs. Following strong vestibular stimulation, such as that caused by an earthquake, heightened sensory vigilance may activate a fight-or-flight response. While sensory reweighting facilitates adaptation, it could be maladaptive, leading to persistent or exaggerated perceptions of motion even in the absence of actual movement [15, 19]. In hypersensitive individuals, even minimal aftershocks may be perceived as significant.

**Prediction errors and mismatch processing:** Earthquakes generate discrepancies between expected and actual sensory input, disrupting the brain’s internal models of motion and spatial orientation. These prediction errors may contribute to prolonged sensations of motion or postural instability [20].

**Psychophysiological interplay:** Anxiety and hypervigilance may amplify somatic sensations and impair vestibular compensation, sustaining SMS perception [21].

**Central sensitization and cortical plasticity:** Repetitive or intense vestibular input during earthquakes may cause maladaptive neuroplastic changes in the cortical regions responsible for spatial orientation. Functional imaging studies have implicated the temporoparietal junction and insular cortex in persistent dizziness and spatial disorientation [22, 23].

**Comments 16.** Explore the implications of research findings for post-earthquake mental health service provision.

**Response 16:** Added discussion on mental health support after earthquakes.

Line 287

This study underscores the significance of addressing anxiety and fear elicited by SMS — a form of state anxiety specifically associated with earthquake-related symptoms. Accordingly, post-disaster surveys should be designed not merely as research instruments but as tools that provide direct, meaningful benefits to participants. Such surveys should aim to collect data and serve as gateways to support systems by offering accurate health information, screening for psychological symptoms, and facilitating access to mental health services. Achieving this goal requires coordinated efforts among researchers, clinicians, and local authorities [28]. Remote consultation systems involving professionals outside the disaster-affected area may further help alleviate the burden on local healthcare infrastructure while maintaining continuous support for those affected.

**Comments 17.** Include a scatter plot or heat map illustrating the relationship between earthquake intensity and SMS. Develop a visual representation of the multivariate risk prediction model.

Create a timeline chart showing symptom evolution over time.

**Response 17:** We appreciate your valuable suggestions. We have added a heatmap showing the seismic intensity of earthquakes and the incident rate of SMS (Fig 2.), as well as a timeline chart showing the time until symptoms disappear (Fig 5.), to enable clear visualization.

Line 125
The incidence rates by seismic intensity and region are shown in Fig 2.

**Reviewer #3:** The effect of a large earthquake on people’s psychology and their daily life is the main topic which needs to pay more attentions. In this paper, the authors cast an internet survey on self-motion sensation by a questionnaire after the large 2024 7.6 earthquake in Noto Peninsula. 1000 responses have been received finally. They do some statistical work on the data and get some valuable results.

However, there are some uncertainties in the work:
**Comments 1.** Firstly of all, for a statistical investigation, the number of the 1000 samples in this time is too small to get a convincing result.

**Response 1:** We chose a sample size that could be collected in 1 day to increase the accuracy of the cross-sectional study. While 1,000 may appear small, this exceeds the size in most prior studies on post-earthquake dizziness and is reasonable given the urgent disaster context.

Line 84

To maintain internal consistency across participants, the response window was limited to 24 h, and 1,000 responses were obtained.

**Comments 2.** Secondly, for a research paper, the investigating method (or technique) and the aim (or to solve a problem) are important. However, we cannot find any relevant contents in this paper.

**Response 2**: We appreciate this helpful comment. In response, we have clarified the study’s aim and methodology in the Introduction and emphasized the clinical implications in the Conclusion. In particular, we now highlight the relevance of state anxiety to persistent symptoms and the importance of psychological screening in future post-disaster health strategies.

Line 53

Many individuals have reported brief episodes of dizziness occurring in the absence of actual aftershocks [8,9]. However, it remains unclear whether this phenomenon constitutes a pathological condition. Moreover, the symptoms reported varied across different studies [2,8,9,15], and its prognosis and the necessity of medical intervention remain partially elucidated.

Line 63

This study aimed to identify the characteristics of new-onset swaying or dizziness —defined here as self-motion sensation (SMS) — experienced after a major earthquake. We also sought to determine the factors associated with persistent symptoms or disturbance in daily life. Identifying these factors may inform early intervention strategies and support services for individuals at risk of persistent symptoms or functional impairment.

Line 91

The primary outcomes were the characteristics of new-onset swaying or dizziness experienced after the earthquake: impact on daily life and symptom prognosis. The secondary outcomes included individual characteristics associated with disturbance in daily life and symptom persistence.

Line 307

SMS after a major earthquake resolves spontaneously and may be a non-pathological response in most people. However, in this study conducted 2 months after the 2024 Noto Peninsula earthquake, although a causal relationship cannot be inferred owing to the cross-sectional study design, state anxiety was found to be significantly associated with persistent symptoms and disturbance in daily life. Our findings highlight the significance of early identification and support for individuals at risk of symptom persistence, as well as the importance of longitudinal studies in clarifying the potential progression of SMS to chronic vestibular disorders such as PPPD. Future post-disaster health strategies should incorporate psychological screening and support systems to mitigate long-term impacts and improve recovery outcomes.

**Comments 3.** So this paper seems like a survey report instead of a research paper. Not mention that all figures are too simple and with low resolution. So a large amount of samples and scientific information are needed to support to this work if it is to publish in the future.

**Response 3:** We have revised the manuscript to enhance its scientific value. In this study, we clarified risk factors (e.g., state anxiety) that may require medical and mental health care, and provided data that can inform the design of future longitudinal studies. We have also improved all figures with higher resolution and added more informative visualizations (Figs 2, 5) to better support the findings.
