# Supplementary material for: Characteristics of self-motion sensation after major earthquakes: An internet survey
Source: PLoS One. 2025 Sep 17;20(9):e0330450. doi: 10.1371/journal.pone.0330450 (PMC12443278; doi:10.1371/journal.pone.0330450)
Supplement: Attachment — Submitted filename: Response to Reviewers0723. docx.docx [file pone.0330450.s007.docx]

We sincerely thank the reviewers for their insightful comments and constructive suggestions, which have greatly enhanced the clarity and rigor of our manuscript. Below, we provide a point-by-point response to each comment, along with the corresponding revisions made in the manuscript.

1. Selection Bias and Representativeness
Comment: While the authors have included Table 1 showing the age distribution of participants and an expanded discussion on selection bias, the analysis of how this bias may influence the generalizability of findings could be slightly more detailed.
Suggestion: Consider quantifying the representativeness of the sample (e.g., by comparing participant demographics to census data or using representativeness indices). This would provide a more concrete assessment of the impact of selection bias.
**Response 1**: We appreciate this valuable suggestion. In response, we have quantified the representativeness of our sample using the Index of Dissimilarity (ID), a standard metric used to evaluate the deviation between a study sample and the general population. The calculated ID was 31.4%, indicating a moderate discrepancy—specifically, the underrepresentation of individuals aged ≥70 years and overrepresentation of those aged 50–69 years.

Line122
The Index of Dissimilarity between the sample and the target population was 31.4%.

Line280
Our sample was biased toward younger individuals compared with the general population of the affected area; consequently, the representativeness of our sample was assessed using the Index of Dissimilarity, which yielded a value of 31.4%, indicating a moderate discrepancy between the sample and the target population. Specifically, individuals aged ≥70 years were underrepresented, while those aged 50–69 years were overrepresented.

2. Recall Bias
Comment: The authors acknowledged recall bias due to the 2-month delay in data collection and justified this timing. However, the potential variability in symptom reporting due to recall bias remains underexplored.
Suggestion: Briefly discuss how recall bias may have influenced specific findings (e.g., over-reporting or under-reporting of SMS) and whether this bias may differ between younger and older participants. A sentence or two in the discussion would suffice.

**Response 2**: We thank the reviewer for this important point. To address this, we have added a concise but more nuanced discussion of potential age-related recall bias. Previous studies have shown that older adults tend to underreport negative emotional experiences in retrospective surveys, whereas younger adults tend to recall such experiences with greater emotional intensity, particularly when the recall interval is prolonged (references have been added, 26: Charles et al., 2016; 27: Junghaenel et al.,2021).

Line 289
Furthermore, because the survey was conducted 2 months after the earthquake, and most SMS symptoms resolve within 2 weeks, recall bias is a potential concern. Similarly, age-related differences in recall processes may have influenced symptom reporting. Older adults tend to underreport negative experiences in retrospective surveys, whereas younger adults are more likely to recall such experiences with increased intensity, particularly over longer recall periods [26,27]. Such age-related recall biases may explain some of the differences in disturbance of daily life owing to SMS between age groups observed in this study.

3. Operational Definition of SMS
Comment: The revised definition of SMS is clearer and better distinguishes it from dizziness. However, the explanation of how participants distinguished SMS from aftershocks could be slightly expanded.
Suggestion: Consider adding a brief sentence clarifying whether participants were explicitly instructed to differentiate SMS from aftershocks (e.g., through self-checks or online earthquake reports).
**Response 3**: The questionnaire asked, “have you felt swaying or dizziness even though there was no actual earthquake at that time?” As the reviewer pointed out, we assume that participants visually confirmed the swaying of objects around them or checked online real-time earthquake information and determined that it was not an aftershock. The following sentence has been added.

Line 234
While sensory reweighting facilitates adaptation, it could be maladaptive, causing persistent or exaggerated perceptions of motion even in the absence of actual movement [14, 18]. The questionnaire asked, “have you felt swaying or dizziness even though there was no actual earthquake at that time?” It was assumed that participants distinguished between aftershocks and SMS by checking for swaying of surrounding objects and earthquake information on the Internet. However, in hypersensitive individuals, even minimal aftershocks may be perceived as significant.

4. Language and Clarity
Comment: While the authors have improved the manuscript's language, a few sentences remain overly complex or slightly ambiguous.

**Response 4**: In response to the reviewers' suggestions, we have requested English proofreading and revised the text for clarity and conciseness. For example, we have rephrased “feeling anxious or scared owing to SMS” to “feelings of anxiety or fear induced by SMS” to improve precision and academic tone. In addition, the descriptions in Figure 3 have been revised to enhance clarity and brevity.

The other revised parts are highlighted in the manuscript, as listed below.

Line 38
Emotional responses, particularly anxiety or fear triggered by self-motion sensation, play crucial roles in daily life impact and symptom persistence.

Line 44
Additionally, dizziness accompanied by headaches, along with insomnia and heightened nervousness, has been described as a common neuropsychiatric symptom following such events [5,6].

Line 99
Participants provided informed consent by checking a consent box on the study website.
Line 129
**Fig 2. Incidence rate of the swaying or dizziness sensation in the absence of an earthquake, based on seismic intensity and region**

Line 136
The Noto earthquake on January 1 had intensities of 4–7, 4–5, and 3–5 in Ishikawa, Toyama, and Fukui Prefectures, respectively. In Ishikawa Prefecture, over 50% of participants reported experiencing dizziness or a swaying sensation in the absence of an earthquake, noting it was their first time.

Line 144
The sensations occurred while participants were in stationary positions, such as sitting, standing, or lying down, and were not associated with body or head movements (Fig 3A). Half of the participants who experienced SMS reported feelings of anxiety or fear in response to the sensation (Fig 3C).

Line 152
(B) Duration of SMS: symptom duration was mostly <1 min (82.7%) and rarely >5 min. (C) Feelings of anxiety or fear induced by SMS: Half of the participants with SMS reported anxiety or fear induced by the sensation.
Line 162
SMS disturbed the daily lives of 18.5% of the participants.

Line 188
Similarly, Miwa et al. reported a 42.2% prevalence of post-earthquake dizziness following the 2016 Kumamoto earthquake [8].

Line 194
SMS involved brief episodes of body sway lasting less than one minute, typically occurring while sitting or standing still, independent of head movement, and distinct from rotational vertigo.

Line 257
Factors associated with SMS-related daily life disturbances overlap with PPPD risk factors.

We have removed the following reference, as only the abstract was available and we were unable to access the full text.

*Tevzadze N, Shakarishvili R. Vertigo syndromes associated with earthquake in Georgia. Georgian Med News. 2007;148-149: 36-39.*
